# Synergistic Remediation of Eutrophic Rural Pond Water Using Submerged Macrophytes and *Daphnia magna*

**DOI:** 10.3390/plants14203136

**Published:** 2025-10-11

**Authors:** Haoyu Cao, Chunxue Zhang, Bo Yang, Liyuan Liu, Jiarui Wang, Xiangqun Zheng

**Affiliations:** 1 Agro-Environmental Protection Institute, Ministry of Agriculture and Rural Affairs, Tianjin 300191, China; 2 Institute of Environment and Sustainable Development in Agriculture, Chinese Academy of Agricultural Sciences, Beijing 100081, China

**Keywords:** rural ponds, eutrophication, *D. magna*, submerged plants, combined remediation

## Abstract

Eutrophication in rural ponds has become a widespread environmental concern, particularly in regions affected by agricultural irrigation. This study proposes an innovative Submerged Macrophytes–*Daphnia magna* combined remediation technology, aiming to synergistically improve water quality in naturally eutrophic ponds. Experimental water was sourced from rural ponds with preserved natural phytoplankton and bacterial communities. Treatments included low- and high-density *D. magna*, two submerged macrophyte species (*Myriophyllum aquaticum* and *Ceratophyllum demersum*), and their combinations. Results showed that combined treatments had no significant effect on pH but improved water transparency by up to 63.6% and significantly increased dissolved oxygen. Nutrient removal was notably enhanced in combined groups, with low-density *D. magna* + *M. aquaticum* achieving TN, TP, and NO_3_^−^-N reductions of 56.1%, 63.2%, and 58.7%, respectively. Both macrophytes effectively mitigated NH_4_^+^-N accumulation caused by *D. magna*, with *M. aquaticum* showing stronger inhibition. Furthermore, low-density *D. magna* reduced phytoplankton density, cyanobacteria density, chlorophyll-a, and microcystins by 74.8%, 80.3%, 68.9%, and 71.2%, respectively. This combined bioremediation approach demonstrates high ecological efficiency, scalability potential, and practical applicability for rural pond restoration.

## 1. Introduction

In recent years, water environmental pollution in China has become increasingly severe, with water eutrophication emerging as a critical and urgent ecological challenge [1]. One of the most characteristic and destructive manifestations of eutrophication is the frequent occurrence of algal blooms [2]. These blooms are predominantly driven by *Cyanobacteria* proliferation, which not only rapidly deteriorates water quality through the release of algal metabolites but also causes mass mortality of aquatic organisms such as fish and submerged macrophytes, ultimately leading to severe disruption of aquatic ecosystem structure and function [3,4,5]. This issue is particularly pronounced in stagnant water bodies, such as rural ponds, where limited water exchange greatly reduces the ecosystem’s self-purification capacity. When external nutrient inputs, especially nitrogen and phosphorus, increase, the risk of eutrophication escalates dramatically, often exceeding the natural resilience and recovery capability of the ecosystem [6].

Typical cases include recurrent *Cyanobacterial* blooms in major lakes and reservoirs such as Taihu Lake, Chaohu Lake, Dianchi Lake, and the Yuqiao Reservoir in Tianjin. These blooms not only disrupt the balance and stability of aquatic ecosystems but also release harmful secondary metabolites such as microcystins, posing significant threats to aquatic life, animals, and human health [7,8]. Eutrophication-driven aquatic ecosystem degradation manifests as marked declines in biodiversity, impaired water functions, and exacerbated water scarcity, thereby seriously threatening regional ecological security and public health, as well as causing substantial economic losses [9].

In response to these challenges, bioremediation technologies have garnered widespread attention due to their cost-effectiveness, environmental compatibility, and minimal secondary pollution risk [10]. Aquatic plants, including submerged and floating species, can directly absorb excess nitrogen and phosphorus from the water column, thereby reducing nutrient loads and providing habitat for aquatic fauna [11]. Concurrently, microorganisms such as bacteria and fungi play crucial roles in degrading organic pollutants and lysing algal cells through diverse biochemical pathways [12]. Moreover, fish and other filter-feeding zooplankton contribute to bioremediation by grazing on algae and aquatic vegetation, improving water clarity and mitigating excessive biomass accumulation.

Extensive studies have demonstrated the potential of individual organisms in alleviating eutrophication. For instance, Zhao et al. [13] extracted allelopathic substances from the submerged macrophyte *Ceratophyllum demersum* under laboratory conditions, which significantly inhibited the growth and disrupted the morphology of *Chlorella vulgaris* in co-culture experiments. Zhang et al. [6] reported that both *Vallisneria natans* and *C*. *demersum* markedly suppressed algal proliferation when co-cultured with the green algae *Cladophora*. Chislock et al. [14] documented that *Daphnia pulicaria* could tolerate microcystins and substantially reduce cyanobacterial biomass in bloom-affected waters. Li et al. [15] isolated strain H10 from *Exiguobacterium*, which indirectly lysed *Microcystis aeruginosa* cells by secreting organic acids. Wojtal-Frankiewicz et al. [16] evaluated the roles of *Daphnia longispina* and *Dreissena polymorpha* in remediation, finding that although mussels (*D. polymorpha*) increased the total phosphorus concentration of ambient water via excretion, zooplankton overall contributed to bloom control.

Despite these advances, single-species approaches often fail to meet the complex remediation demands of rural pond ecosystems, which typically bear multiple pollutant loads, advanced eutrophication stages, and impaired ecological functions. Thus, there is an urgent need to develop and optimize integrated bioremediation technologies that leverage the complementary strengths of multiple organisms. Current research on plant–microbial remediation predominantly focuses on the synergistic effects of aquatic plants and immobilized bacterial strains [17]. Studies have shown that ecological theories emphasizing multi-trophic interactions have attracted growing interest, proposing that interactions among diverse aquatic species can be harnessed to suppress harmful algal blooms and restore ecosystem balance [17,18].

Empirical studies confirm that submerged macrophytes effectively reduce nitrogen and phosphorus concentrations, inhibit algal growth, and enhance water transparency [19]. Zooplankton such as *Daphnia magna* exert top-down grazing pressure on phytoplankton populations, thereby improving water quality [20]. Recent studies have explored the combined use of biomanipulation and submerged macrophytes to control algal blooms and remediate eutrophic water bodies. Applying two or more aquatic organisms in combination has attracted attention due to potential synergistic effects. For example, Li et al. [21] used the freshwater mussel *Anodonta woodiana* and *Vallisneria natans* in a mesocosm, showing that the combined system significantly reduced chlorophyll and nutrient concentrations, while mussels promoted plant growth, increasing shoot height by 24.9% compared to monoculture. Guo [22] constructed surface-flow artificial wetlands using mussels and snails with *Potamogeton crispus*, achieving TN and TP removal of 82.8% and 98.5%, respectively; NH_4_^+^-N removal was mainly due to sediment disturbance and microbial enhancement. Huo et al. [23] introduced *Daphnia magna* into Shihu Lake followed by submerged macrophytes, resulting in significantly lower TN, NH_4_+-N, and TP concentrations compared to controls. Ma et al. [24] combined *V. natans* with *D. magna* in ponds, greatly reducing nutrients, improving transparency, and promoting plant growth, with wet weight increasing 740% versus 470% in monoculture.

Despite these advances, most studies have focused on mesocosms or specific sites, with limited assessment under variable pond conditions. Moreover, the combined remediation effects of *D. magna* and submerged macrophytes remain insufficiently explored, and their practical potential in eutrophic rural ponds has yet to be fully assessed. To address this gap, the present study simulates eutrophic rural pond water under controlled conditions to quantify the synergistic effects of submerged macrophytes and *D. magna* on nutrient removal, algal suppression, and plant growth, providing a theoretical basis for future field applications. Accordingly, we propose an integrated bioremediation strategy tailored to rural eutrophic pond conditions. By utilizing raw pond water containing indigenous microbial and algal communities, this approach aims to better reflect realistic ecological settings and offer valuable insights for practical restoration efforts.

Specifically, a novel remediation system combining submerged macrophytes (*Myriophyllum aquaticum* and *Ceratophyllum demersum*) and the zooplankton *D. magna* was designed to control eutrophication via dual mechanisms: nutrient uptake by aquatic plants and algal grazing by zooplankton. This strategy not only facilitates pollutant removal but also promotes the re-establishment of balanced aquatic ecosystems [25]. Laboratory co-culture experiments simulating natural spring and summer bloom conditions systematically evaluated the individual and combined effects of these organisms on eutrophic water quality, nutrient dynamics, phytoplankton community structure, and microcystin concentrations. The findings are intended to provide a scientific foundation for developing effective and sustainable bioremediation technologies applicable to rural water bodies.

## 2. Materials and Methods

### 2.1. Test Materials

#### 2.1.1. Test Water

In this study, the raw water was taken from a eutrophication pond polluted by planting irrigation in Ninghe County, Tianjin (N 39°33′, E 117°82′). The water characteristics are listed in Table 1.

The sampled water was filtered with a no. 13 zooplankton net to remove original microzooplankton, then diluted with a sterilized BG-11 (4%) culture solution in a 1:1 ratio. After dilution, the concentrations of TN and TP were consistent with those in the raw pond water, while the native phytoplankton and bacterial communities were reduced to half of their original abundance. The diluted water was then used as the test water in this experiment to preserve an appropriate living space for organisms.

#### 2.1.2. Tested Submerged Plants

*M. aquaticum* and *C. demersum* were purchased from a flower market in Tianjin Before the experiment, they were cultured in BG-11 (4%) solution for 15 days to adapt to the experimental environment, and then well-grown plants of similar size were then selected for test.

#### 2.1.3. Tested *D. magna*

*D. magna* was purchased from the Institute of Applied Ecology, Chinese Academy of Sciences in Shenyang and its cultivation enlarged in the laboratory before the test. The *D. magna* was placed into a 1 L flask containing the test water, and acclimated in an artificial climate chamber for more than 60 days at a temperature of 25 °C, light intensity of 1200 lux, light-to-dark ratio of 12:12 h, feed 20 mL of the Scenedesmus obliquus solution daily and change the water every other day. Before the experiment, active and healthy offspring of similar size were selected through a filter with a 2 mm pore size, and cultured in pure water for 24 h to empty their intestines as much as possible before the experiment [26].

### 2.2. Experiment Designs

The test barrels were constructed of polyvinylchloride (PVC) with a 30 cm diameter and a 30 cm height. Before the experiment, the barrels were sterilized with ethanol (75%) and repeatedly rinsed with pure water. Fluorescent lamps were used to simulate daylight in spring and summer. The light intensity was 1200 ± 100 lux, light-to-dark ratio was 12:12 h. The test water body was thoroughly mixed and divided into each test barrel, with the initial water content of 20 L. The remaining water was used for the detection of various indicators, which was taken as the initial value of 0 d. The experiments were conducted at a constant temperature of 25 °C, chosen based on pre-experiments and literature as a representative temperature that allows normal physiological activity of *Daphnia magna* and submerged macrophytes, and approximates the average summer temperature of the sampled pond. To maintain this temperature, a heating rod was installed in each test barrel, and thermometers were set to continuously monitor the water temperature, ensuring that the experimental water remained stable throughout the study. The test time lasted for 20 d. There was a total of nine groups (Table 2) with three replicates. Group K was the control treatment; no animals or plants were added. The effects of different densities of *D. magna* on eutrophic water were studied in groups A and B. Groups C and D were added with the same wet weight (60 g) of different submerged plants to study the effects on eutrophic water. The four groups E, F, G and H were “Submerged Macrophytes-*D. magna*” combinations, with low/high-density *D. magna* combined with two plants, respectively.

The samples were taken from each flask at 0, 2, 5, 10, 15 and 20 days for determination, and the pH, DO, water transparency, TN, TP, NH_4_^+^-N, NO_3_^−^-N, Chla, Phytoplankton density, Cyanobacteria density, Microcystin concentration were measured. At the end of the experiment (20 days), the *D. magna* density and the wet weight of the plants were measured. Sampling was conducted between 8:00–9:00 a.m. The water level was recorded after each sampling to reduce errors, and the evaporation is made up by distilled water before the next sample [27].

### 2.3. Tested Methods

#### 2.3.1. Determination of Water Quality

The pH and dissolved oxygen (DO) were measured in situ using a dual-input multi-parameter digital analyzer (HQ40d, Hach Company, Loveland, CO, USA). Water samples were collected from different layers within the experimental setup, thoroughly mixed, and then measured, with the resulting values used as the representative pH and DO for each treatment.

The transparency of pond water was determined using a Secchi disk, following standard procedures.

A 20 mL water sample was collected, and nutrient concentrations, including total nitrogen (TN), total phosphorus (TP), ammonia-nitrogen (NH_4_^+^-N), and nitrate-nitrogen (NO_3_^−^-N), were determined using an Auto Analyzer 3 system (SEAL Analytical GmbH, Norderstedt, Germany) based on flow injection analysis. TN was measured by alkaline potassium persulfate digestion followed by ultraviolet spectrophotometry, TP was determined using the molybdenum-antimony anti-spectrophotometric method, NH_4_^+^-N was quantified via the Nessler’s reagent colorimetric method, and NO_3_^−^-N was assessed using the phenol disulfonic acid colorimetric method. All analyses were performed in triplicate to ensure accuracy and reproducibility.

#### 2.3.2. Determination of Phytoplankton

To determine phytoplankton density, chlorophyll-a (Chl-a) concentration, and microcystin (MC) content, plants in each test bucket were carefully removed, and the water was gently stirred with a glass rod before collecting a 250 mL water sample. Of this, 100 mL was fixed with 10 mL Lugol’s solution and concentrated to 30 mL for microscopic examination. Another 100 mL was used for Chl-a determination, and the remaining water was used for MC analysis.

For phytoplankton counting, 1 mL of the concentrated sample was pipetted into a 0.1 mL counting chamber and examined under an Olympus microscope at 400× magnification. Three subsamples per sample were counted, and the average was taken as the phytoplankton density. Chl-a was measured using a fluorometer (Model 10-005, Turner Designs Hydrocarbon Instruments, Inc., Fresno, CA, USA). Chlorophyll was extracted in acetone at 20 °C in darkness for 24 h. Microcystin concentrations were determined using an enzyme-linked immunosorbent assay (ELISA) following the manufacturer’s instructions (Abraxis, Warminster, PA, USA) [28].

#### 2.3.3. *D. magna* Density and Plants Wet Weight

A 1 L water sample was collected and fixed with 3% formaldehyde, then stirred with a glass rod and concentrated to 50 mL. A 1 mL aliquot was placed in a counting chamber and examined under a microscope at 100× magnification. Each sample was counted three times, and the average value was recorded as the *D. magna* density.

Submerged plants from each test bucket were carefully removed, and surface water was gently blotted with filter paper. The plants were weighed three times using an electronic balance, and the average value was recorded as the wet weight.

### 2.4. Data Analysis and Processing

The reduction rate of physical and chemical indexes is calculated as follows:

$$\mathrm{Reduction}\;\mathrm{rate}\;=\;\frac{\left(C_{x}\;-{\;C}_{0}\right)}{C_{0}}\;\times \;100\%$$

where C_0_—The initial concentration of an indicator;

$C_{x}$—The concentration of this indicator on day x.

All indicators were Origin 9.0 mapping, and the data were expressed in the form of mean ± standard deviation of three parallel test buckets. SPSS 24.0 was used for statistical analysis. The difference significance of indicators was tested by one-way/ANOVA, and Tukey’s Honestly Significant Difference (HSD) test (*p* < 0.05) was used for pairwise comparisons following a one-way ANOVA to identify significant differences between treatment groups.

## 3. Results and Discussion

### 3.1. Effects of Different Daphnia magna Densities on Water Quality and Algal Suppression

#### 3.1.1. Effects on pH, Dissolved Oxygen (DO), and Water Transparency

**Figure 1:**
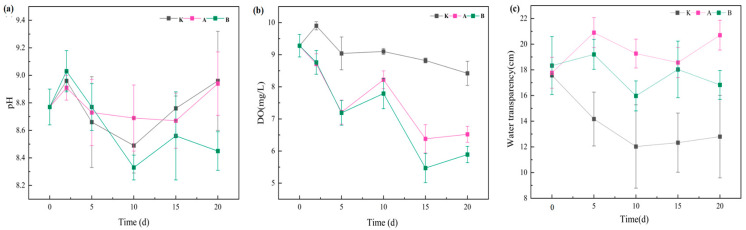
Temporal changes in pH, dissolved oxygen (DO), and water transparency across treatments. Note: (**a**) pH; (**b**) Dissolved oxygen (DO); (**c**) Water transparency. Different uppercase letters indicate different *D. magna* densities: A, 25 ind/L; B, 100 ind/L. Data are presented as mean ± standard deviation (*n* = 3).

Temporal variations in pH, DO, and water transparency under different treatments are presented in Figure 1. All treatments maintained an alkaline environment (pH 8.1–9.4). Compared with the control (K group), neither low-density (A group) nor high-density (B group) *D. magna* treatments significantly altered pH (*p* > 0.05). However, from days 10 to 20, the pH in the B group showed a slight decline, likely due to enhanced respiration under high-density conditions, generating CO_2_ that reacted with water to form carbonic acid, thereby lowering pH [29]. Additionally, intensive grazing suppressed algal photosynthesis and reduced CO_2_ uptake; the release of organic acids and CO_2_ through excretion and organic matter decomposition, along with acid production during nitrification, weakened the buffering capacity of the water, further contributing to pH reduction.

DO concentrations exhibited distinct temporal patterns. During the initial phase (0–5 days), both the A and B groups showed a marked decrease in DO, with reductions significantly greater than that of the control (*p* < 0.05), indicating a pronounced effect of *D. magna* respiration on oxygen consumption. Between days 5 and 10, DO levels rebounded to peak values, with the A group (8.22 ± 0.277 mg/L) exceeding the B group (7.79 ± 0.474 mg/L), possibly due to a stronger compensatory effect of algal photosynthesis under low-density conditions. In the later phase (10–20 days), DO levels in all treatments declined continuously, reaching 6.25 ± 0.25 mg/L in the A group and 5.89 ± 0.26 mg/L in the B group by day 20, both significantly lower than in the K group (8.42 ± 0.38 mg/L) (*p* < 0.05). The more severe hypoxic condition in the high-density group reflects the cumulative effect of population density on oxygen depletion.

At the start of the experiment, no significant differences in transparency were observed among treatments (*p* > 0.05), suggesting that *D. magna* required an acclimation period to establish effective grazing activity. After day 5, transparency increased significantly in both the A and B groups (*p* < 0.05), indicating that *D. magna* effectively improved water quality by grazing on suspended particles, detritus, and algae [22]. Throughout the experimental period, transparency in the A group remained higher than in the B group, although the difference was not statistically significant (*p* > 0.05). This suggests that further increasing *D. magna* density does not significantly enhance transparency improvement. The likely explanation is that at low density, individuals exhibit higher grazing efficiency and maintain a more stable algae–*Daphnia* balance, whereas in high-density conditions, although total grazing increases, sediment resuspension, accumulation of excretory products, and shifts in algal community structure [24] may offset the benefits, indicating the presence of a saturation threshold in grazing effects.

#### 3.1.2. Variations in Nutrient Concentrations

**Figure 2:**
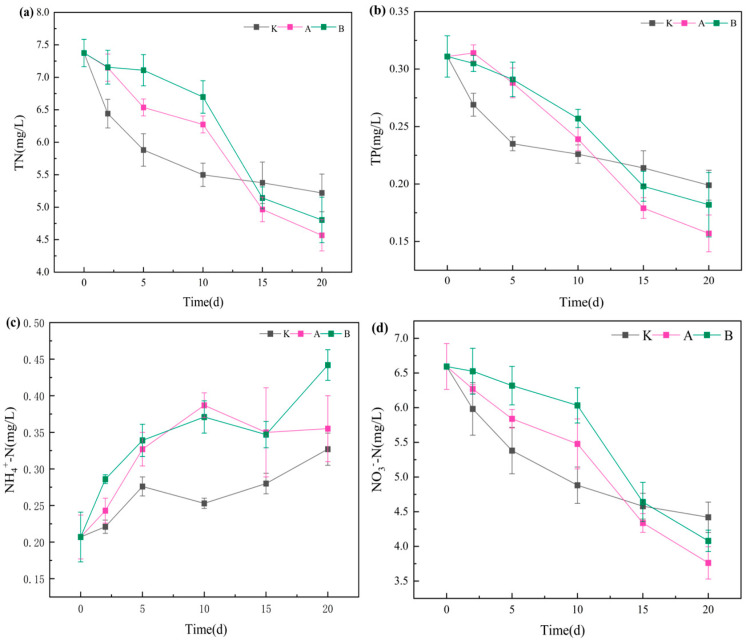
Nutrient concentrations (TN, TP, NO_3_^−^-N, NH_4_^+^-N) during the 20-day experiment. Note: (**a**) Total nitrogen (TN); (**b**) Total phosphorus (TP); (**c**) Ammonium nitrogen (NH_4_^+^-N); (**d**) Nitrate nitrogen (NO_3_^−^-N). Data are presented as mean ± standard deviation (*n* = 3).

Temporal changes in total nitrogen (TN), total phosphorus (TP), nitrate nitrogen (NO_3_^−^-N), and ammonium nitrogen (NH_4_^+^-N) are shown in Figure 2. In all treatments, TN, TP, and NO_3_^−^-N concentrations decreased over time, reaching their lowest levels by day 20. No significant differences (*p* > 0.05) were observed between the control (K group) and the *D. magna* treatments (A and B groups) in terms of TN, TP, and NO_3_^−^-N concentrations or their reduction rates. During the first 15 days, TN, TP, and NO_3_^−^-N concentrations in the *D. magna* treatments were slightly higher than in the control, whereas by the end of the experiment, the control exhibited higher concentrations, indicating that *D. magna* exerted a certain nutrient-reducing effect.

NH_4_^+^-N showed a distinct pattern: concentrations in all groups increased throughout the experiment. By day 20, NH_4_^+^-N in the B group (0.442 ± 0.02 mg/L) was significantly higher than in the A group (0.355 ± 0.05 mg/L) and the K group (0.327 ± 0.02 mg/L) (*p* < 0.05), while no significant difference was found between A and K. This suggests that high-density *D. magna* may promote NH_4_^+^-N accumulation via excretion and decomposition of carcasses, thereby offsetting their potential nutrient removal benefits [24].

These findings highlight the dual role of *D. magna* in aquatic ecosystems. On the one hand, they assimilate nutrients by grazing on phytoplankton and particulate matter [30], indirectly reducing nitrogen and phosphorus concentrations by lowering phytoplankton biomass. On the other hand, their metabolic activity and decomposition release NH_4_^+^-N. In the early phase (0–15 days), higher nutrient concentrations in the treatments may have resulted from the rupture of algal cells during grazing, temporarily releasing dissolved nutrients. In the later phase (15–20 days), as populations stabilized, assimilation and sedimentation processes lowered nutrient concentrations below those in the control. The marked NH_4_^+^-N accumulation in the B group likely resulted from increased metabolic excretion and higher mortality rates under high-density conditions. This reflects a “density threshold effect” in zooplankton-mediated nutrient regulation—beyond a critical density, excretory products can become a secondary nutrient source [29].

#### 3.1.3. Effects on Phytoplankton and Microcystins

**Figure 3:**
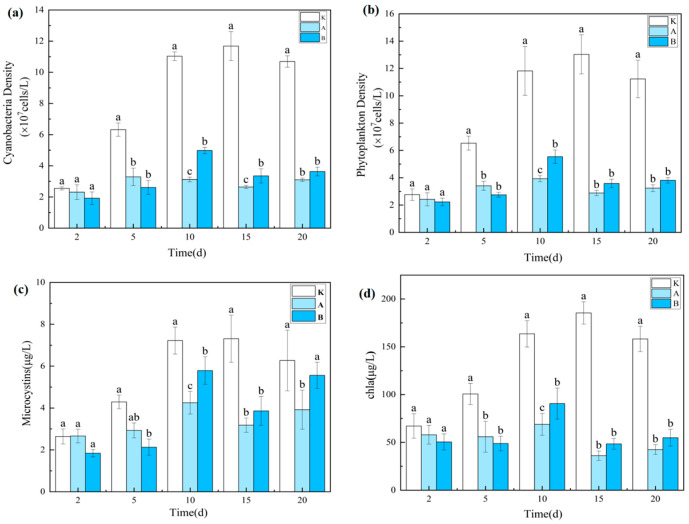
Phytoplankton density, cyanobacterial biomass, and chlorophyll-a levels under *D. magna* treatments. Note: (**a**) Cyanobacteria density; (**b**) Phytoplankton density; (**c**) Microcystin concentration; (**d**) Chlorophyll a concentration. Different lowercase letters indicate significant differences among treatments at the same time point (*p* < 0.05, *n* = 3).

Chlorophyll-a (Chl-a), a key pigment in photosynthesis, is closely correlated with phytoplankton abundance [31] and is widely used as a proxy for phytoplankton biomass. In this study, Cyanophyta (cyanobacteria) consistently dominated the phytoplankton community (>85%), resulting in similar temporal trends for phytoplankton density, cyanobacterial biomass, and Chl-a concentration (Figure 3).

As a herbivorous cladoceran, *D. magna* can substantially reduce phytoplankton abundance through grazing. By day 5, phytoplankton density, cyanobacterial biomass, and Chl-a concentrations in both the A and B groups were significantly lower than in the control (*p* < 0.05), confirming strong initial grazing pressure. However, by day 20, no significant difference was detected between A and B (*p* > 0.05), and the B group exhibited higher values than the A group, indicating that increased density did not further enhance algal suppression.

Microcystin (MC) concentrations fluctuated in an “increase–decrease–increase” pattern under *D. magna* influence. On day 10, MC levels peaked in the A and B groups (4.25 ± 0.54 μg/L and 5.79 ± 0.66 μg/L, respectively), whereas the control peaked later, on day 15 (7.31 ± 1.13 μg/L) (*p* < 0.05). By day 20, MC concentrations in the A group (3.92 ± 0.93 μg/L) were significantly lower than in the control (6.27 ± 1.45 μg/L) (*p* < 0.05), while the B group (5.56 ± 0.63 μg/L) showed no significant difference from the control (*p* > 0.05). Although the high-density group exhibited strong initial grazing effects, excessive excretion and carcass decomposition in the later phase likely promoted nutrient regeneration and secondary algal growth, causing MC concentrations to rebound.

This pattern indicates a nonlinear relationship between *D. magna* density and algal suppression. In the early phase (0–5 days), both treatments achieved significant reductions in phytoplankton due to direct grazing. Over time, however, high-density conditions in the B group likely intensified competition, reducing per capita grazing efficiency, while metabolic byproducts (e.g., NH_4_^+^) and carcass decomposition facilitated algal regrowth. MC, as a cyanobacterial metabolite, is positively correlated with cyanobacterial abundance [32,33] and can negatively impact *D. magna* growth and reproduction [34]. In this study, MC accumulation in the B group (5.56 μg/L) may have inhibited *D. magna* feeding activity. Conversely, the A group maintained a more stable ecological balance, with nutrient cycling and algal regrowth rates in dynamic equilibrium, and MC levels (3.92 μg/L) below inhibitory thresholds, thus sustaining longer-term algal control.

MC can accumulate in aquatic organisms [35], but *D. magna* are small-bodied and have low tolerance, making them more susceptible to high MC concentrations [36]. Given the high cyanobacterial density in this study (>10^7^ cells/L), significant mortality occurred in both treatments. By day 20, *D. magna* densities had declined to (12.7 ± 5.5) ind/L in A and (41.8 ± 13.7) ind/L in B, representing large decreases from initial stocking densities (25 ind/L and 100 ind/L, respectively), with greater reductions in the high-density treatment due to stronger intraspecific competition. Upon death, *D. magna* can release accumulated MC back into the water, further weakening suppression in the later phase.

In summary, *D. magna* effectively suppressed phytoplankton and MC in the early phase, with low-density treatments providing more stable and prolonged effects. High-density treatments, despite strong initial suppression, experienced diminished efficacy over time due to biological stress and nutrient release effects.

### 3.2. Effects of Two Submerged Macrophytes on Water Quality

#### 3.2.1. Variations in pH, Dissolved Oxygen (DO), and Water Transparency

**Figure 4:**
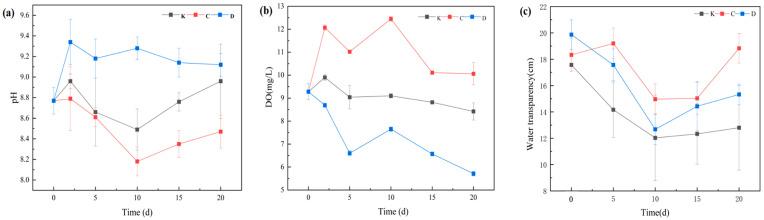
Microcystin concentrations in different treatment groups over time. Note: (**a**) pH; (**b**) Dissolved oxygen (DO); (**c**) Water transparency. Different uppercase letters indicate the type of submerged macrophytes: C, 3 g/L *M. aquaticum*; D, 3 g/L *C. demersum*. Data are presented as mean ± standard deviation (*n* = 3).

Figure 4 illustrates the temporal variations in pH, DO, and water transparency in treatments with *Myriophyllum aquaticum* (C group) and *Ceratophyllum demersum* (D group). In the C group, pH ranged from 8.18 to 8.79, initially decreasing and then increasing during the experimental period, with a final reduction from 8.77 ± 0.13 to 8.47 ± 0.16. In contrast, the D group exhibited an overall increase followed by a decline, with pH rising from 8.77 ± 0.13 to 9.12 ± 0.11 by the end of the experiment. Neither change was statistically significant (*p* > 0.05). These contrasting pH patterns likely reflect differences in the dynamic balance between photosynthesis and respiration in the two macrophytes. The initial pH increase in the D group may result from rapid CO_2_ uptake during intense photosynthetic activity, while the subsequent decrease could be attributed to enhanced respiration or organic matter decomposition as biomass increased. Conversely, the initial pH decline in the C group may relate to respiration dominance or organic acid excretion, whereas the later rise may indicate enhanced photosynthesis or buffering via the carbonate system. Morphological differences, carbon acquisition strategies, and impacts on microbial communities may also contribute to these variations.

DO concentrations exhibited pronounced temporal fluctuations (*p* < 0.05), with the C group maintaining significantly higher DO levels than the D group throughout the experiment (*p* < 0.05). This can be attributed to the more developed root system and stronger photosynthetic capacity of *M. aquaticum*, which enhance oxygen release into the water column.

In terms of transparency, no significant differences were observed among groups during the early phase (day 2; *p* > 0.05). However, from day 5 onward, transparency significantly increased in all treatment groups compared with the control (*p* < 0.05). Compared with the control, water transparency in the C and D groups increased, which was mainly attributed to the submerged macrophytes improving water clarity by absorbing nutrients, competing for light, and releasing allelopathic compounds that inhibited phytoplankton growth. Transparency did not significantly differ between the C and D groups (*p* > 0.05), suggesting comparable short-term efficacy in enhancing water clarity.

#### 3.2.2. Changes in Nitrogen and Phosphorus Concentrations

**Figure 5:**
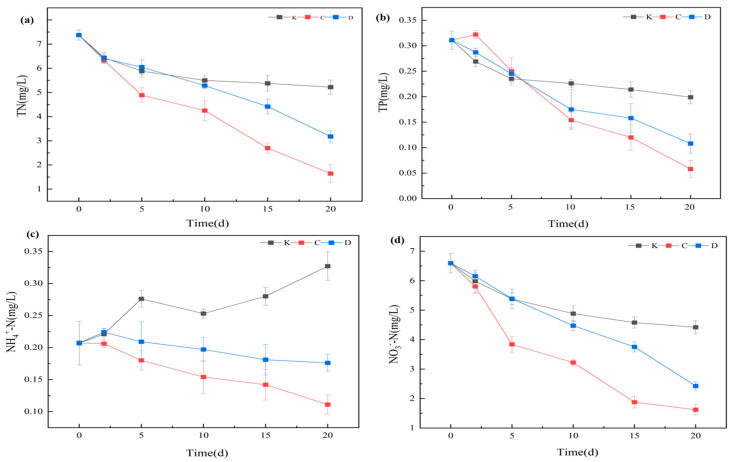
Nutrient removal efficiency of *H. verticillata* and *E. canadensis*. Note: (**a**) Total nitrogen (TN); (**b**) Total phosphorus (TP); (**c**) Ammonium nitrogen (NH_4_^+^-N); (**d**) Nitrate nitrogen (NO_3_^−^-N). Data are presented as mean ± standard deviation (*n* = 3).

As shown in Figure 5, both macrophyte treatments markedly reduced total nitrogen (TN), total phosphorus (TP), nitrate nitrogen (NO_3_^−^-N), and ammonium nitrogen (NH_4_^+^-N) concentrations over time. In both the C (*M. aquaticum*) and D (*C. demersum*) groups, nutrient concentrations declined steadily, reaching their lowest values by day 20. The removal efficiencies in the C group reached 77.68% for TN, 81.35% for TP, 75.26% for NO_3_^−^-N, and 46.38% for NH_4_^+^-N, all significantly higher than the control (*p* < 0.05, *n* = 3). This demonstrates that both species effectively suppress phytoplankton growth by depleting waterborne nutrients [37].

Comparative analysis indicated that the C group achieved significantly greater nutrient removal than the D group (*p* < 0.05), with particularly pronounced efficiency in NH_4_^+^-N removal. Specifically, NH_4_^+^-N in the C group decreased from 0.207 ± 0.034 mg/L to 0.111 ± 0.015 mg/L (46.38% removal), whereas in the D group it declined to only 0.176 ± 0.013 mg/L. These findings are consistent with Xing et al. [38], who reported that systems combining *M. aquaticum* and *C. demersum* exhibited superior nitrate and total phosphorus removal compared with other plant assemblages.

Notably, NH_4_^+^-N concentrations in the control group (K) showed a continuous accumulation, increasing from 0.207 ± 0.034 mg/L to 0.327 ± 0.022 mg/L (*p* < 0.05), while the C and D groups achieved reductions of 46.38% and 14.96%, respectively. At the end of the experiment, NH_4_^+^-N levels in both macrophyte treatments were significantly lower than in the control and in zooplankton treatments (A and B groups; *p* < 0.05). This demonstrates that submerged macrophytes can effectively remove not only nitrate but also ammonium, with *M. aquaticum* showing superior performance, making it particularly valuable for eutrophic water restoration [39,40].

The enhanced nutrient removal capacity of *M. aquaticum* is likely due to its extensive root system and high nitrate reductase activity, which enable more efficient nutrient uptake. Additionally, organic acids secreted in the rhizosphere stimulate denitrifying bacteria, fostering a more active nitrogen transformation microenvironment. Furthermore, the oxygen gradients established within its canopy favor coupled nitrification–denitrification processes, while maintaining an optimal pH range (7.2–8.5) that supports the growth and metabolic activity of associated microbial communities.

#### 3.2.3. Dynamics of Phytoplankton Communities and Microcystin-LR (MC-LR) Concentrations

At the end of the experiment, key eutrophication indicators—including cyanobacterial density, total phytoplankton density, and chlorophyll-a concentration—were significantly reduced (*p* < 0.05) in both submerged macrophyte treatments compared with the control group (K). Temporal analysis revealed that *Myriophyllum aquaticum* (C group) exhibited the strongest inhibitory effect on phytoplankton. By day 10, cyanobacterial density, total phytoplankton density, and chlorophyll-a concentration in the C group were reduced by 51.68%, 52.45%, and 47.55%, respectively, relative to the control, with suppression significantly greater than that observed in the *Ceratophyllum demersum* treatment (D group; *p* < 0.05). The *M. aquaticum* may inhibit phytoplankton growth via the release of species-specific allelopathic compounds.

The stronger phytoplankton suppression by *M. aquaticum* is partly attributable to its greater nutrient removal capacity compared with *C. demersum*. In addition to nutrient uptake, submerged macrophytes can inhibit phytoplankton through light and space competition, as well as allelopathic interference [41].

**Figure 6:**
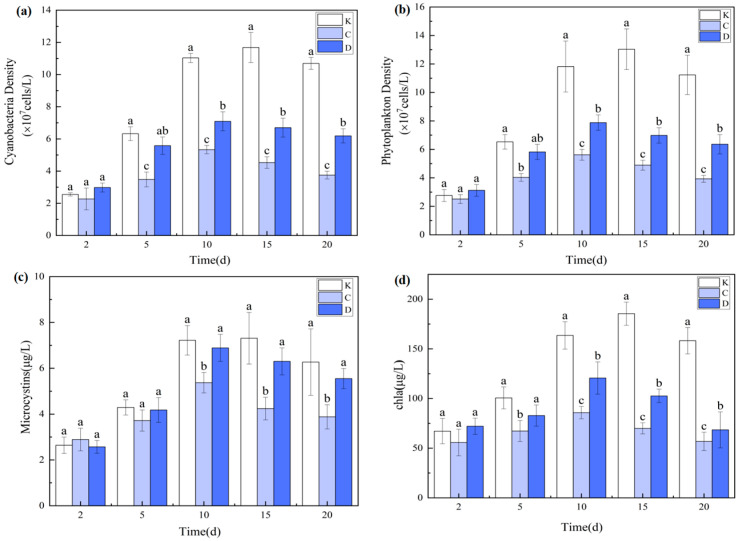
Phytoplankton and microcystin reduction by submerged macrophytes. Note: (**a**) Cyanobacteria density; (**b**) Phytoplankton density; (**c**) Microcystin concentration; (**d**) Chlorophyll a concentration. Different lowercase letters indicate significant differences among treatments at the same time point (*p* < 0.05, *n* = 3).

Microcystin-LR (MC-LR) concentrations (Figure 6) showed significant differences between the two macrophyte treatments at the end of the experiment (*p* < 0.05). The lowest MC-LR concentration occurred in the C group (3.88 ± 0.42 µg/L), which was significantly lower than both the control (6.27 ± 0.58 µg/L) and the D group (5.55 ± 0.51 µg/L; *p* < 0.05). No significant difference was observed between the D and control groups (*p* > 0.05). This indicates that *M. aquaticum* not only effectively suppresses phytoplankton growth but may also reduce MC-LR levels via adsorption or degradation processes, whereas *C. demersum* did not exhibit significant toxin removal capacity.

The relatively poor MC-LR reduction observed in the D group may be due to two factors. First, *C. demersum* exerted weaker suppression on phytoplankton and cyanobacteria densities than *M. aquaticum*. Second, the lysis and death of cyanobacterial cells can release large amounts of MC-LR into the water [42]. Zhang [35] reported that *C. demersum* can release allelochemicals such as α-methylphenylethanol and adipic acid diesters, which disrupt algal cell membranes and induce cell lysis, thereby releasing intracellular MC-LR. In the present study, the slower growth rate and lower MC-LR adsorption capacity of *C. demersum* may explain why, despite inhibiting cyanobacterial density to some extent, it exhibited limited ability to reduce MC-LR concentrations.

These findings provide novel theoretical support for the use of submerged macrophytes, particularly *M. aquaticum*, as an ecological strategy to control cyanobacterial blooms and mitigate microcystin pollution.

### 3.3. Effects of Two Submerged Macrophytes with Different Daphnia magna Densities on Water Quality

#### 3.3.1. Variations in pH, Dissolved Oxygen (DO), and Water Transparency

**Figure 7:**
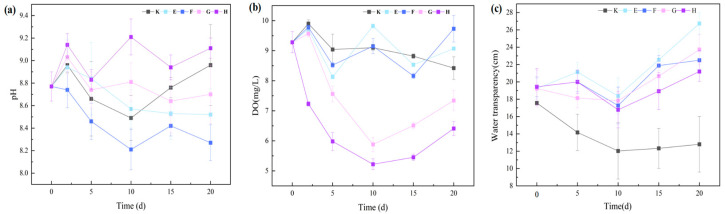
DO, pH, and transparency in combined macrophyte–*D. magna* treatments. Note: (**a**) pH; (**b**) Dissolved oxygen (DO); (**c**) Water transparency. Different uppercase letters represent different combinations of submerged macrophytes and *D. magna* densities: E, 25 ind/L *D. magna* + 3 g/L *M. aquaticum*; F, 100 ind/L *D. magna* + 3 g/L *M. aquaticum*; G, 25 ind/L *D. magna* + 3 g/L *C. demersum*; H, 100 ind/L *D. magna* + 3 g/L *C. demersum*, and data are presented as mean ± standard deviation (*n* = 3).

As shown in Figure 7, The combined submerged *macrophyte*–*Daphnia magna* treatments exhibited a pronounced stabilizing effect on water pH. Throughout the cultivation period, pH values in all treatment groups fluctuated within a narrow range (8.27–9.11), with no significant differences among groups (*p* > 0.05). By the end of the experiment, the highest pH was observed in group H (9.11 ± 0.09), whereas groups E (8.52 ± 0.23), F (8.27 ± 0.16), and G (8.70 ± 0.26) showed varying degrees of decline. This pH stability is attributable to the ecological balance between oxygenic photosynthesis by submerged macrophytes and respiratory CO_2_ release by *D. magna*.

DO dynamics during the first 10 days differed significantly among treatments (*p* < 0.05). In the *C. demersum*–*D. magna* combinations (G and H), DO concentrations dropped sharply by 36.64% and 43.75%, respectively—significantly greater declines than those in group F (1.42%) and the control (1.94%). In contrast, DO in group E increased by 5.82%. These divergent DO patterns may be explained by differences in macrophyte biomass and thus photosynthetic oxygen production, shifts in phytoplankton community structure, and high *D. magna* densities exceeding the oxygen supply capacity of the system through respiration.

All macrophyte–*D. magna* combinations significantly improved water transparency compared with the control (*p* < 0.05) from day 5 to day 20. By the end of the experiment, transparency improvements ranked as follows: E (108.83%) > G (85.39%) > F (75.78%) > H (65.63%). Low-density *D. magna* treatments (E and F) produced significantly greater transparency enhancements than their high-density counterparts (G and H) (*p* < 0.05), likely due to reduced sediment resuspension caused by lower grazing-induced disturbance.

Transparency enhancement was primarily driven by multiple synergistic pathways: nutrient removal by submerged macrophytes from both the water column and sediments, thereby limiting phytoplankton growth potential [24]; direct inhibition of phytoplankton through shading and allelopathic compounds; and the removal of phytoplankton and detritus by *D. magna*, which markedly reduced turbidity [43]. These findings confirm that appropriately balanced submerged macrophyte–*D. magna* combinations can significantly improve water clarity through complementary ecological mechanisms, providing a valuable technical reference for eutrophic water restoration.

#### 3.3.2. Nitrogen and Phosphorus Removal Characteristics and Synergistic Mechanisms

**Figure 8:**
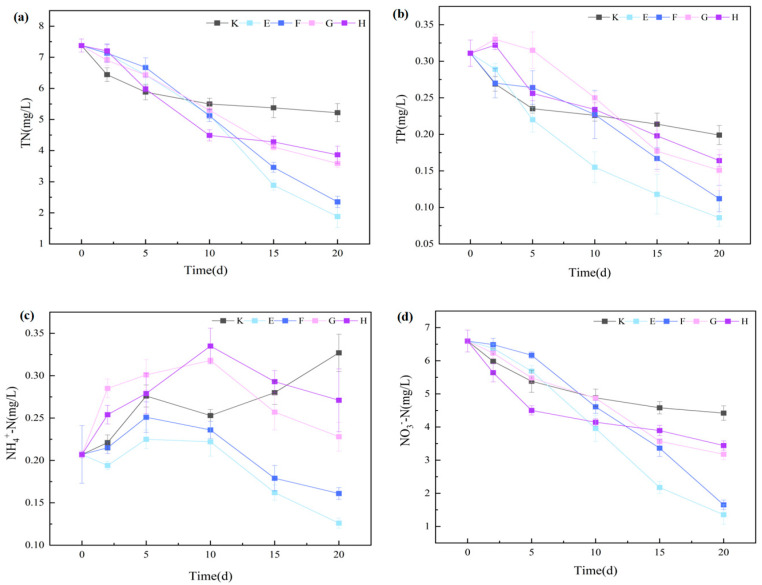
Nitrogen and phosphorus removal by different co-remediation groups. Note: (**a**) Total nitrogen (TN); (**b**) Total phosphorus (TP); (**c**) Ammonium nitrogen (NH_4_^+^-N); (**d**) Nitrate nitrogen (NO_3_^−^-N). Data are presented as mean ± standard deviation (*n* = 3).

As shown in Figure 8, concentrations of total nitrogen (TN), total phosphorus (TP), nitrate nitrogen (NO_3_^−^-N), and ammonium nitrogen (NH_4_^+^-N) in all treatments exhibited a pronounced temporal decline over the experimental period (*p* < 0.05). By day 20, the *Myriophyllum aquaticum*–*Daphnia magna* combinations (E and F) achieved significantly higher nutrient removal efficiencies than the *C. demersum* combinations (G and H) (*p* < 0.05). Specifically, TN removal rates in E and F reached 74.49% and 68.12%, respectively, exceeding those of G (51.34%) and H (47.61%) by 45.09% and 43.08%. TP removal rates were 72.35% (E) and 63.99% (F), compared with 51.45% (G) and 47.27% (H). These results indicate that *M. aquaticum*–*D. magna* systems possess superior nutrient control capacity.

NH_4_^+^-N removal at the end of cultivation further highlighted the advantage of optimal grazer density: the low-density *D. magna*–*M. aquaticum* group (E) removed 39.13% of NH_4_^+^-N, significantly outperforming the high-density counterpart (F, 22.22%) (*p* < 0.05). This effect is likely due to reduced metabolic excretion at lower grazer densities, which limits secondary nitrogen release, combined with the high NH_4_^+^-N affinity of *M. aquaticum* compensating for grazer excretion. NO_3_^−^-N removal followed a pattern consistent with TN, further confirming the synergistic purification effect of this combined system.

Overall, plant species and zooplankton density exerted decisive influence on nutrient removal efficiency in the macrophyte–zooplankton system. The low-density *D. magna*–*M. aquaticum* configuration achieved the best overall performance. Compared with *D. magna* alone, all macrophyte–grazer combinations significantly enhanced TN, TP, NO_3_^−^-N, and NH_4_^+^-N removal, demonstrating a clear synergistic effect. While *D. magna* has limited direct nutrient removal capacity, submerged macrophytes were the primary drivers [44], with grazers indirectly enhancing purification by improving water transparency and promoting macrophyte growth. Excessive grazer density, however, may elevate nitrogen loads via increased mortality or excretion, thereby diminishing synergy.

Ecologically, *D. magna*–*M. aquaticum* combinations—particularly low-density grazers—formed a mutually beneficial system. Macrophyte stands provide habitat and refuge for zooplankton [45], and *D. magna* biomass is positively correlated with plant volume inhabited (PVI) (R = 0.47, *p* = 0.0001) [46]. By grazing microalgae and facilitating particulate sedimentation, *D. magna* supports macrophyte photosynthesis and oxygen release, which in turn benefits grazer growth [47]. Macrophytes also directly limit algal proliferation through nutrient and inorganic carbon uptake [48]. These findings provide a robust technical basis for optimizing macrophyte–zooplankton configurations in eutrophic water restoration.

Although nutrient concentrations reached their lowest measured values by day 20 (Figure 2, Figure 6 and Figure 8), they might not have fully stabilized and could continue to change over a longer period. The 20-day experimental duration was determined based on preliminary experiments. Future studies should further investigate these potential long-term variations to better understand the dynamic nutrient transformation processes in the combined *Daphnia magna*—macrophyte system

#### 3.3.3. Synergistic Suppression of Phytoplankton and Microcystins

**Figure 9:**
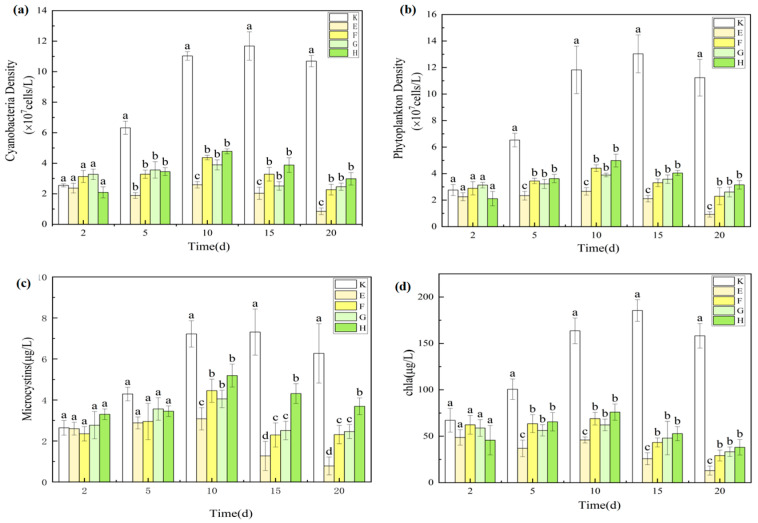
Algal inhibition and MC suppression in all treatment combinations. Note: (**a**) Cyanobacteria density; (**b**) Phytoplankton density; (**c**) Microcystin concentration; (**d**) Chlorophyll a concentration. Different lowercase letters indicate significant differences among treatments at the same time point (*p* < 0.05, *n* = 3).

As shown in Figure 9, all combined treatments significantly suppressed phytoplankton compared with the control. Time-series monitoring revealed that from day 5 onward, total phytoplankton density, cyanobacterial density, and chlorophyll-a concentrations in all treatments were significantly lower than in the control (*p* < 0.05), with suppression persisting until the end of the experiment (day 20). The low-density *Daphnia magna*–*Myriophyllum aquaticum* group (E) exhibited the strongest inhibitory effect, reducing phytoplankton biomass by 91.81% ± 2.34% relative to the control, significantly outperforming all other treatments (*p* < 0.05).

Microcystin (MC) analysis (Table 2) at day 20 showed a distinct concentration gradient: E (0.78 ± 0.438 μg/L) < F (2.31 ± 0.446 μg/L) < G (2.46 ± 0.339 μg/L) < H (3.69 ± 0.41 μg/L). MC concentrations in E were significantly lower than in all other treatments (*p* < 0.05), representing an 87.56% reduction compared with the control, and 66.23%, 68.29%, and 78.86% reductions compared with F, G, and H, respectively. These results highlight the superior toxin control capacity of the E treatment.

Between-group comparisons indicated that *D. magna*–*M. aquaticum* combinations (E, F) were markedly more effective in suppressing phytoplankton and MC than *D. magna*–*Ceratophyllum demersum* combinations (G, H) (*p* < 0.05), underscoring the critical role of macrophyte species in determining restoration performance. This superiority may be attributed to the vigorous growth of *M. aquaticum* in this study, which enhanced its ability to suppress phytoplankton through competition for light and space as well as allelopathic compound release. In contrast, *C. demersum* displayed slower growth and lower MC adsorption capacity under the experimental conditions.

At the end of cultivation, low-density grazer combinations (E, G) consistently outperformed their high-density counterparts (F, H), a trend also observed in single-grazer treatments. In E and G, *D. magna* density increased by 35% and 18% from initial levels, whereas F and H experienced declines of 42% and 55%, respectively. Submerged macrophytes provide refuge for grazers, reducing intraspecific competition, and at low densities, *D. magna* populations reproduce parthenogenetically, exhibiting higher reproduction rates and feeding activity, thereby enhancing phytoplankton suppression. Conversely, high grazer densities trigger a shift to sexual reproduction due to competitive stress, reducing feeding efficiency and ecological control capacity. Moreover, elevated excretion at high densities accelerates N and P cycling, indirectly promoting phytoplankton growth [37].

Collectively, these findings confirm that the low-density *D. magna*–*M. aquaticum* combination (E) achieved the highest performance in phytoplankton suppression, MC removal, and overall water quality improvement. With phytoplankton and MC reduction rates of 82.3% and 87.5%, respectively, this configuration offers a robust and scalable ecological restoration strategy for eutrophic waters.

## 4. Conclusions

This study utilized water from rural ponds impacted by agricultural irrigation, pretreated to retain indigenous phytoplankton and bacterial communities, thereby ensuring ecological relevance for practical eutrophic water restoration. Results indicated that *Daphnia magna*, *Myriophyllum aquaticum*, *Ceratophyllum demersum*, and their combined submerged macrophyte–*D. magna* treatments had no significant effect on pH, but consistently improved water transparency, with combined treatments showing the greatest increases. Compared with *D. magna* alone, combined treatments significantly enhanced dissolved oxygen (DO) concentrations, particularly in the low-density *D. magna* + *M. aquaticum* group.

Nutrient removal performance varied markedly among treatments. *D. magna* alone had negligible effects on total nitrogen (TN), total phosphorus (TP), or nitrate nitrogen (NO_3_^−^-N), and higher zooplankton densities increased ammonium nitrogen (NH_4_^+^-N). In contrast, submerged macrophytes significantly reduced TN, TP, NO_3_^−^-N, and NH_4_^+^-N, with *M. aquaticum* outperforming *C. demersum*. Combined treatments achieved the most pronounced nutrient reductions, with the low-density *D. magna* + *M. aquaticum* group attaining TN, TP, and NO_3_^−^-N removal rates of 56.1%, 63.2%, and 58.7%, respectively.

Algal control was also enhanced under combined treatments. *D. magna* significantly decreased phytoplankton density, cyanobacterial density, chlorophyll-a, and microcystin concentrations, with greater inhibition at low densities (25 ind/L) than at high densities (100 ind/L). By day 20, microcystin levels in the high-density group were comparable to the control. Both macrophytes suppressed algal growth, with *M. aquaticum* showing superior inhibitory capacity; however, *C. demersum* did not significantly reduce microcystin levels.

Overall, this work demonstrates the synergistic role of submerged macrophytes and *D. magna* in nutrient removal and harmful algal suppression in eutrophic waters, identifies the optimal combination for restoration (low-density *D. magna* + *M. aquaticum*), and provides a scientific basis for the ecological management of rural pond ecosystems. In summary, our results demonstrated that the combined application of *Daphnia magna* and submerged macrophytes effectively improved water quality in eutrophic rural ponds by reducing nutrient concentrations, increasing transparency, and suppressing phytoplankton growth. This experiment was, however, limited by its short duration and single-site source water under controlled conditions, and future studies should therefore focus on multi-site, long-term field trials to validate the broader applicability and ecological stability of this combined remediation approach.

## Figures and Tables

**Table 1:** Initial parameters of test water.

Indexes.	Values	Indexes	Values
pH	8.77	Nitrate nitrogen (mg/L)	6.59
Dissolved oxygen (mg/L)	9.28	Chlorophyll a (μg/L)	68.59
Water transparency (cm)	17.20	Phytoplankton density (cells/L)	2.80 × 10^7^
Total nitrogen (mg/L)	7.38	Cyanobacteria density (cells/L)	2.52 × 10^7^
Total phosphorus (mg/L)	0.31	Microcystin concentration (μg/L)	2.77
Ammonia-nitrogen (mg/L)	0.21		

Note: Dissolved oxygen (DO), Water transparency (WT), Total nitrogen (TN), Total phosphorus (TP), Ammonia-nitrogen (NH_4_^+^-N), Nitrate nitrogen (NO_3_^−^-N), Chlorophyll a (Chla), Phytoplankton density (PD), Cyanobacteria density (CD), Microcystin concentration (MC).

**Table 2:** Experimental design of different treatments.

Groups		*D. magna*	*M. aquaticum*	*C. demersum*
Control K		/	/	/
*D. magna*	A	25 ind·L^−1^	/	/
	B	100 ind·L^−1^	/	/
submerged plants	C	/	3 g·L^−1^	/
	D	/	/	3 g·L^−1^
*D. magna* + submerged plants	E	25 ind·L^−1^	3 g·L^−1^	/
	F	100 ind·L^−1^	3 g·L^−1^	/
	G	25 ind·L^−1^	/	3 g·L^−1^
	H	100 ind·L^−1^	/	3 g·L^−1^

## Data Availability

Data are contained within the article.
